# Evaluation of Loop-Mediated Isothermal Amplification Assay for the Detection of* Pneumocystis jirovecii* in Immunocompromised Patients

**DOI:** 10.1155/2015/819091

**Published:** 2015-11-19

**Authors:** Preeti Singh, Sundeep Singh, Bijay Ranjan Mirdha, Randeep Guleria, Sanjay Kumar Agarwal, Anant Mohan

**Affiliations:** ^1^Department of Microbiology, All India Institute of Medical Sciences, Ansari Nagar, New Delhi 110029, India; ^2^Department of Pulmonary Medicine and Sleep Disorders, AIIMS, Ansari Nagar, New Delhi 110029, India; ^3^Department of Nephrology, AIIMS, Ansari Nagar, New Delhi 110029, India

## Abstract

*Pneumocystis* pneumonia (PCP) is one of the common opportunistic infection among HIV and non-HIV immunocompromised patients. The lack of a rapid and specific diagnostic test necessitates a more reliable laboratory diagnostic test for PCP. In the present study, the loop-mediated isothermal amplification (LAMP) assay was evaluated for the detection of* Pneumocystis jirovecii.* 185 clinical respiratory samples, including both BALF and IS, were subjected to GMS staining, nested PCR, and LAMP assay. Of 185 respiratory samples, 12/185 (6.5%), 41/185 (22.2%), and 49/185 (26.5%) samples were positive by GMS staining, nested PCR, and LAMP assay, respectively. As compared to nested PCR, additional 8 samples were positive by LAMP assay and found to be statistically significant (*p* < 0.05) with the detection limit of 1 pg. Thus, the LAMP assay may serve as a better diagnostic tool for the detection of* P. jirovecii* with high sensitivity and specificity, less turn-around time, operational simplicity, single-step amplification, and immediate visual detection.

## 1. Introduction

*Pneumocystis* pneumonia (PCP) is an opportunistic fungal infection caused by* Pneumocystis jirovecii*. It causes life threatening pneumonia among HIV as well as non-HIV immunocompromised patients. Clinical manifestation of PCP found to be different among HIV and non-HIV immunocompromised patients. PCP is a diagnostic challenge due to nonspecific signs and symptoms. Laboratory diagnosis by microscopy is less sensitive and often inconclusive. In 1990, Wakefield and colleagues [1] first described the molecular detection of* P. jirovecii*. Since then, several molecular assays directed at various gene targets employed for the detection of this organism included the dihydropteroate synthase (DHPS), dihydrofolate reductase (DHFR), internal transcribed spacer (ITS) regions of the rRNA gene, and mt LSU rRNA. Among all the available PCR platforms based assays, nested PCR assay targeting mitochondrial large subunit ribosomal RNA (mt LSU rRNA) was found to be one of the widely employed assays for the detection of* P. jirovecii* [2, 3].

However, the nested PCR assays are bit cumbersome and carryover contamination prone. To overcome such limitations more advanced quantitative real time PCR (qPCR) assays with the added advantage of simultaneous amplification and detection have also been employed in developed countries. Furthermore, the qPCR based assays have additional advantage of higher specificity lesser turnaround time and chances of contamination but require costly equipment. Therefore, they are not feasible in general clinical laboratories settings, especially in developing countries.

Loop-mediated isothermal amplification (LAMP) recently developed by Notomi et al. is a rapid, simple, and cost effective method of DNA amplification [4]. It is an isothermal technique, based upon autocycling strand displacement DNA synthesis by* Bst* DNA polymerase. LAMP primers consisted of six sets of primers comprising two outer (F3 and B3), two inner (FIP and BIP), and two loop primers (FL and BL). These sets of primers recognize eight distinct regions of the target sequence. The FIP primer consisted of F2 and the complementary strand of F1 (F1c). The BIP primer consisted of B2 and the complementary strand of B1 (B1c) and thus ensures high specificity of the assay. Additionally, LAMP ensures higher yield of target gene, that is, 10^9^, in less than an hour in a simple incubator. Further, one-step amplification reaction followed by visual detection based on turbidity has contributed to LAMP assay as an attractive alternative to PCR/qPCR platforms based assays for routine microbiological diagnostic tool [5]. An added advantage of LAMP assay is that the positive amplifications can be effortlessly detected and distinguished from the negative PCP samples without the need of gel electrophoresis. This is achieved by simply observing the turbidity of the end product in the reaction tube. Besides this, colorimetric analysis can also be done by adding DNA intercalating dyes like SYBR Green I and Propidium iodide to the end products in the reaction tube.

Since the introduction of LAMP assay, it has been extensively used and evaluated with other nucleic acid amplification techniques for the detection of various pathogens, namely,* Legionella* spp. [6],* Mycobacterium* spp. [7],* Paracoccidioides* [8], dengue virus [9],* Salmonella* spp. [10], Methicillin-Resistant* Staphylococcus aureus* [11],* Listeria monocytogenes* [12],* Plasmodium* spp. [13], and* Entamoeba histolytica* [14].

Thus, the aim of the present study was to evaluate the applicability of a LAMP assay for the detection of* Pneumocystis jirovecii*.

## 2. Material and Methods

### 2.1. Study Details

#### 2.1.1. Study Design

Prospective study was designed to evaluate loop-mediated isothermal amplification (LAMP) assay for the detection of* Pneumocystis jirovecii* in immunocompromised patients.

#### 2.1.2. Study Subjects

One hundred eighty-five immunocompromised adult (>18 years) patients with the high index of clinical suspicion of PCP were enrolled in the study. These 185 patients consisted of 64 HIV seropositive patients with CD4^+^ T cells count below 200 cells/mm^3^ and 121 non-HIV immunocompromised patients (immunosuppressive drugs and chemotherapy). Among 121 non-HIV immunocompromised patients, 66 were postrenal transplant recipients, 20 with malignant disorders, and 35 with other immunocompromised states.

#### 2.1.3. Study Site

The study was conducted in Department of Microbiology, All India Institute of Medical Sciences (AIIMS), New Delhi.

#### 2.1.4. Duration of Study

Study was conducted over a period of 3 years (2010–2013).

#### 2.1.5. Number of Samples

A total of 185 respiratory samples (nonduplicate) were obtained, including 135 bronchoalveolar lavage fluids (BALF) and 50 induced sputa (IS) (Table 1). All these samples were obtained from outpatients and inpatients of Departments of Pulmonary Medicine, Nephrology and Medical Oncology, of our tertiary care referral hospital.

#### 2.1.6. Inclusion Criteria

Immunocompromised patients above 18 years of age with high index of clinical suspicion of PCP were included in the study.

#### 2.1.7. Exclusion Criteria

Individuals not fulfilling the inclusion criteria and/or not willing to participate in the study were excluded.

#### 2.1.8. Informed Consent Procedure

Informed consent was taken from all the patients enrolled under study. The demographic profile and clinical and radiological characteristics of all the patients were taken into account prospectively (data not shown). Ethical approval for this study was obtained from the Institutional Ethics Committee.

### 2.2. Sample Processing and DNA Extraction

For extracting DNA induced sputum samples were initially treated with 6.5 mM dithiothreitol (DTT), a mucolytic agent, and then both BALF and IS samples were centrifuged. 50 *μ*L of the pellet was used for preparing a smear for GMS staining. DNA extraction was done using a Qiagen DNeasy tissue kit (Qiagen Inc., Valencia, CA, USA) following manufacturer's instructions with some modifications. 200 *μ*L of pellet was lysed in 200 *μ*L of lysis buffer containing 50 mM KCl, 15 mM Tris-HCl (pH-8.3), 0.5% NP-40, and 500 *μ*g of proteinase K followed by incubation at 56°C for 45 minutes [15]. To the above mixture, 200 *μ*L of buffer AL (provided with the kit) was added and the contents were mixed immediately and incubated at 70°C for 10 minutes. After incubation, 200 *μ*L of ethanol (96%) was added and mixed thoroughly by vortexing. The above mixture was applied to the DNeasy minispin columns (provided with the kit) and was centrifuged at 8000 rpm for 1 minute and the step was repeated until the whole mixture was used up. Thereafter, 500 *μ*L of buffer AW1 was added to the column and centrifugation was carried out again at 8000 rpm for 1 minute. This was followed by a second washing with buffer AW2 and centrifugation. To elute the DNA, the column was placed in a microcentrifuge (1.5 mL) tube and elution buffer AE provided with the kit was pipetted into the DNeasy membrane. It was further incubated for 1 minute at room temperature and centrifuged at 8000 rpm for 1 minute. The eluted DNA was stored at −20°C for further studies. DNA extracted from a previously microscopically positive clinical sample was used as positive control and double distilled water was used as a negative control in the study.

### 2.3. Nested PCR Assay

The DNA extracted from all clinical samples was subjected to nested PCR targeting mt LSU rRNA gene, as described previously by Gupta et al. [3]. The PCR products were analyzed by 1.5% agarose gel electrophoresis.

### 2.4. LAMP Assay

LAMP assay was performed using primers specific for 18S rRNA genes of* P. jirovecii* as described previously by Uemura et al. [16]. The specificity of the primers used was reassessed using in silico BLAST for the accession number AB266392. LAMP assay was standardized using DNA from positive control. The LAMP reaction consisted of 12.5 *μ*L reaction mixture (containing 20 mM Tris-HCl (pH 8.8), 10 mM KCl, 8 mM MgSO_4_, 10 mM (NH_4_)_2_SO_4_, 0.1% (v/v) Tween 20, 0.8 M betaine, and 1.4 mM of each deoxynucleoside triphosphate (dNTP), 1.6 *μ*M of each inner primer (FIP and BIP), 0.2 *μ*M of each outer primer (F3 and B3), 2.5 *μ*L primer mix (6.25 *μ*L), 1 *μ*L* Bst* DNA polymerase, and 3 *μ*L DNA), and final volume was made up with double distilled water for 25 *μ*L reaction. The reaction mixture was incubated in a water bath at 65°C for 60 minutes, followed by heating at 85°C for 3 min to terminate the reaction. Positive reaction was detected as turbidity of the reaction mixture observable with the naked eye. Besides this, the results were assessed by adding propidium iodide to the reaction tube after amplification reaction takes place and observed under ultraviolet light. The amplicons were also analyzed by running on 2%  (wt/vol) agarose gel electrophoresis for typical ladder like pattern. To prevent crossover contamination, all the necessary precautions were taken into consideration like different sets of pipettes and different work areas were designated for the extraction of genomic DNA and performing the LAMP assay.

### 2.5. Sensitivity and Specificity of LAMP Assay

To determine the detection limit of the assay, the genomic DNA extracted from the previously microscopically PCP positive sample (positive control) was diluted 10-fold from 100 ng to 100 fg and the amplified products were analyzed visually as well as by 2% (w/v) agarose gel electrophoresis. The specificity of the LAMP assay was assessed using genomic DNA extracted from other respiratory pathogens, including* Streptococcus pneumoniae*,* Haemophilus influenzae*,* Pseudomonas aeruginosa*,* Candida albicans*,* Aspergillus fumigatus*,* Cryptococcus neoformans*,* Cytomegalovirus* (CMV), and* Mycobacterium tuberculosis*.

### 2.6. Statistical Analysis

A *p* value < 0.05 was considered statistically significant. All statistical analyses were carried out using Statistical Package for the Social Sciences (SPSS) software version 20 (SPSS, USA). The McNemar test was used to compare the sensitivity of the LAMP assay with nested PCR. “Receiver operating characteristic (ROC) curve analysis and area under curve (AUC)” were made to determine the clinical sensitivity and specificity of the LAMP assay.

## 3. Results

### 3.1. Results of Microscopy, Nested PCR, and LAMP Assay

Of 185 patients, 12/185 (6.5%) were GMS staining positive and 41/185 (22.2%) were nested PCR positive. However, 49/185 (22.2%) were LAMP positive. These LAMP assay positive samples consisted of 16 patients from HIV seropositive and 33 from non-HIV immunocompromised patients (21 postrenal transplant (PRT) recipients, 4 with malignant disorders, and 8 among other immunocompromised states (Table 2)).

### 3.2. Distribution of Additional 8 Positive Samples by LAMP Assay

Among 8 additional positive samples by LAMP assay 2 were BALF samples from HIV seropositive patients and 6 were sputa from non-HIV immunocompromised patients (Table 3). All* P. jirovecii* positive samples produced luminescence with dye and showed a typical ladder like pattern when amplicons were run on the 2% agarose gel electrophoresis. There was no discordance between the results of microscopy and molecular assay (nested PCR and LAMP assay); that is, all the GMS positives were also positive by nested PCR and LAMP assay.

### 3.3. Sensitivity and Specificity of LAMP Assay

**Figure 1:**
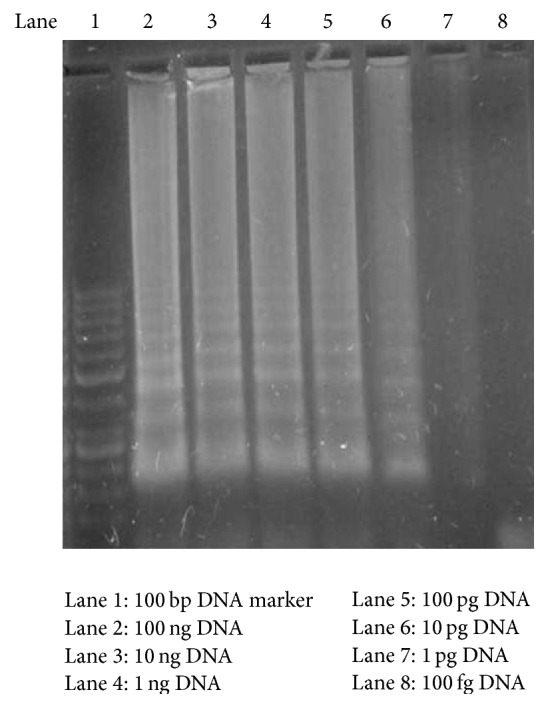
Sensitivities LAMP assay for* Pneumocystis jirovecii* detection (LAMP assay was carried out using 10-fold serially diluted DNA purified from* P. jirovecii* positive samples).

**Figure 2:**
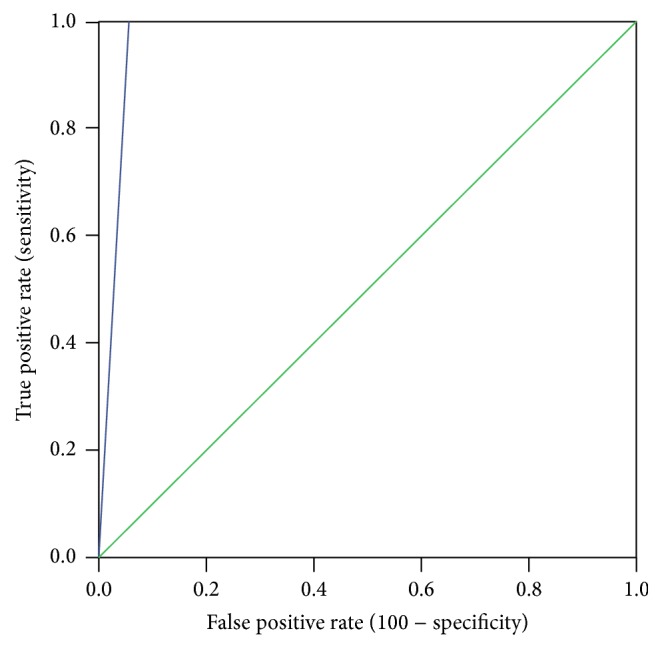
Receiver operating characteristic (ROC) curve for LAMP assay. [The true positive rate (sensitivity) is plotted in function of the false positive rate (100 − specificity). The area under the ROC curve, a measure of how well the LAMP assay distinguishes* P. jirovecii* positive from* P. jirovecii* negative respiratory samples, is = 0.972, 95% CI (0.961–0.983).]

The detection limit of the LAMP assay (analytical sensitivity) was found to be 1 pg of DNA and that is ten times more than that of nested PCR (previously established in the laboratory) in tenfold serial dilution of the 100 ng DNA (Figure 1), thus, showing higher sensitivity of LAMP assay over nested PCR assay. The specificity of the assay was determined to be 100% showing no nonspecific amplification of DNA when tested against genomic DNA of other respiratory pathogens. Clinical sensitivity of the LAMP assay was determined by ROC curve and the area under the curve was 0.972 with a standard deviation of ±0.011 at 95% confidence interval (0.961–0.983) (Figure 2). When the clinical sensitivity of the LAMP assay was compared with that of nested PCR, it was significantly higher with LAMP assay using McNemar test (*p* = 0.008).

## 4. Discussion

Loop-mediated isothermal amplification (LAMP) assay is the boon of the 21st century that amplifies nucleic acid with high specificity, sensitivity, and less turnaround time. In addition, it is a simple, rapid, and cost-effective method, which requires only water bath or heating blocks to perform the assay [4]. Hence, it has been extensively employed as a diagnostic tool for numerous bacterial, viral, and parasitic diseases [6–13]. LAMP assay had also been reported by Uemura et al. for the detection of* P. jirovecii* in both BALF and sputum samples [16]. Subsequently, a number of studies have been conducted on various pathogens and recommended that the LAMP assay has higher sensitivity over other nucleic acid amplification techniques.

Thus, the present study was designed to evaluate the performance of the LAMP assay for the detection of* P. jirovecii* in both BALF and IS samples. The LAMP assay was compared with nested PCR, targeting 18S rRNA gene and mt LSU rRNA gene, respectively, and microscopic examination using GMS staining. In our study, GMS staining was used as it provides a sharp contrast between the background of host cells and organism [17]. Of 185 samples obtained, only 12 BALF samples were positive by GMS staining. The less number of microscopic positive results as compared to nested PCR and LAMP assay might be due to the limitations of GMS staining to detect only cystic form of the parasite and lower parasite burden in clinical samples. Furthermore, there was 100% concordance among the results of GMS positive samples and nested PCR and LAMP assay positive. Of 185 samples 41 samples were detected positive with nested PCR and additional 8 samples were detected positive by LAMP assay. This indicates the higher sensitivity of LAMP assay over nested PCR. The analytical sensitivity of LAMP assay was found to be ten times more than that of nested PCR assay with the detection limit of 1 pg. Sensitivity of the LAMP assay with respect to nested PCR was found to be statistically significant (*p* ≤ 0.05). Furthermore, high specificity of the LAMP assay was also demonstrated by no nonspecific genomic amplification when tested against other respiratory pathogens. Thus, the results of the present study demonstrated that the LAMP assay did not show any cross-reactivity with DNA of other respiratory pathogens and negative controls.

Similar reports of the higher sensitivity of LAMP assay over nested PCR have been reported by Lau et al. and Fallahi et al. in case of* Toxoplasma gondii* [18, 19]. In another study Heidarnejhad and coworkers reported the high sensitivity of the LAMP assay over nested PCR for the detection of* Mycobacterium avium* [20].

In the present study, 8 patients were additional positive by LAMP assay, with clinical and radiological features suggestive of PCP. From these 8 patients, 8 samples were obtained that comprised 2 and 6 BALF and IS, respectively. BAL fluid samples were obtained from HIV seropositive patients with CD4^+^ T cells count more than 100 cells/mm^3^. Both of these patients had received anti-PCP prophylaxis and thus the number of organisms might have decreased [21]. This again reflects the high sensitivity of the LAMP assay over nested PCR.

Among the remaining 6 LAMP assay positive patients, induced sputum samples were obtained. Taking into account that sputum samples contain PCR inhibitors, the same might be encountered in the present study. However,* Bst* DNA polymerase used in LAMP assay is not sensitive to such inhibitors. Thus, it may be speculated that the rate of detection of* P. jirovecii* in both BALF and IS was higher in the case of LAMP assay.

Furthermore, two of these six were having Wagner's diseases with CMV as one of the primary respiratory pathogens; after administration of ganciclovir as anti-CMV drug the signs and symptom of PCP were also resolved. The remaining 4 patients included two PRT recipients, one with sarcoidosis, and one with chronic obstructive pulmonary disease (COPD). The other microbiological investigation demonstrated that the PRT patients were infected with CMV as the primary pathogen. The patient with sarcoidosis was sputum smear positive for* M. tuberculosis* and the COPD patients were having* Candida albicans* as a copathogen. This reflects that these patients might be colonized with* P. jirovecii*. This was an important finding and was in concordance with that of Probst et al. 2000 and Vidal et al. 2006 [22, 23]. Therefore, the diagnostic value of the LAMP assays was superior to nested PCR for both BALF and IS samples among both HIV seropositive and other immunocompromised populations.

To conclude, the LAMP assay is a novel promising technique which is sensitive, specific, rapid, simple, and cost-effective for the detection of* Pneumocystis jirovecii* with the detection limit of 1 pg of DNA. Moreover, the LAMP assay and interpretation of its results require minimal laboratory facilities. Therefore, it could be best employed as a diagnostic tool in resource limiting countries and point of care settings as well.

To the best of our knowledge, this is the first report evaluating LAMP assay with respect to nested PCR for the detection of* P. jirovecii* from this Indian subcontinent.

## Figures and Tables

**Table 1:** Distributions of clinical samples among different patients groups.

Underlying conditions	Number of patients (*n* = 185)	* *Number of samples (*n* = 185)	
		BALF	IS
HIV	64	29	35
PRT	66	62	4
Malignant disorders	20	16	4
Other immunocompromised states^*∗*^	35	28	7
Total	185	135	50

^*∗*^Other immunocompromised conditions: chronic kidney diseases (*n* = 2), pulmonary alveolar proteinosis (*n* = 7), bone marrow transplant recipient (*n* = 4), systemic lupus erythematosus (*n* = 11), post-liver transplant (*n* = 3), Wegner's granulomatosis (*n* = 4), chronic liver disease (*n* = 3), and autoimmune related cirrhosis (*n* = 1).

**Table 2:** Distribution of positive samples by GMS staining, nested PCR, and LAMP assay among different patients groups.

Underlying conditions	Number of samples	Number of positive samples	GMS staining	Nested PCR (mtLSUrRNA)	LAMP assay (18S rRNA)
HIV	64	16	1	14	16
PRT	66	21	10	19	21
Malignancies	20	4	0	4	4
Other immunocompromised conditions	35	8	1	4	8
Total	185	49	12	41	49

**Table 3:** Distribution of additional 8 positive samples by LAMP assay among different patients groups.

Underlying conditions	* *Number of positive samples		
	Positive samples	Nested PCR (mtLSUrRNA)	LAMP assay (18S rRNA)
HIV	2	0	2
PRT	2^*∗*^	0	2
Malignancies	0	0	0
Other immunocompromised conditions	4^*∗*^	0	4
Total	8/8	0	8

^*∗*^Sputum samples.
